# A Complementary Alternative Medicine Questionnaire for Young Adults

**DOI:** 10.4137/imi.s2281

**Published:** 2009-04-07

**Authors:** Christine Patterson, Heather Arthur

**Affiliations:** 1 School of Nursing Faculty of Health Sciences McMaster University, Hamilton, Ontario, Canada; 2 Cardiovascular Nursing Research Faculty of Health Sciences School of Nursing McMaster University, Hamilton, Ontario, Canada

**Keywords:** CAM, adolescent, questionnaire, beliefs

## Abstract

Limited information exists on how adolescents decide to use complementary/alternative medicine (CAM). There are also no instruments specific to CAM, for the young adult population, which makes it difficult to explore knowledge in this area. The purpose of this study was to develop and examine the psychometric properties of the CAM Questionnaire for Young Adults which measures young adults’ attitudes about CAM. Participants for this cross-sectional survey were selected from enrolled undergraduate students at an urban university. Factor analysis identified three subscales: 1) positive beliefs about CAM; 2) environmental influence; and 3) psychological comfort. The scale has good internal consistency (Cronbach’s alpha = 0.79) and shows beginning demonstration of validity. Its use in this sample revealed that young adults who are female and have used CAM in the past for preventing or treating illness have the most positive attitude towards CAM and the greatest likelihood for continued use. The implication that prevention may play a role in young adults’ attitudes about CAM is a potential focus for future research.

## Introduction

Adolescence is a period marked by physical and behavioural changes. It is during this time that adolescents develop skills to make the transition to adulthood by expanding personal decision-making skills, increasing independence and forming their own identities.^1^ As part of this transition process, adolescents and young adults begin to make important decisions related to their health, personal values and lifestyles. Through exposure to different ways of healing, young adults may decide to opt for treatments that fall outside the boundary of mainstream medicine.^2^,^3^

The perceptions of young adults are essential for understanding how factors both within and external to their world may play a role in their health choices. For example, are young adults more likely to seek assistance for health issues from complementary/alternative medicine (CAM) providers rather than their physicians? If so, what contributes to their decisions about who should be delivering the service?

To date, there is little information available on the decision-making processes employed by adolescents and young adults who use CAM. However, knowledge of what influences their likelihood to use CAM is essential for understanding how internal and external factors can play a role in health care decisions of this nature. An understanding of the rationale for health choices will enable practitioners to guide young adults in selecting health care options and will provide a basis for health planning that includes a range of services.^4^ It is also important to increase awareness among nurses, physicians, and allied health care professionals about young adults’ use of CAM therapies, which clinically, could minimize the risk of potentially harmful pharmaceutical-natural substance interactions and increase young adults’ satisfaction with health care service provision.^5^–^7^

Currently there is a lack of research specific to young adults’ use of CAM.^8^ Recommended areas of further study include utilization patterns; the influences of gender and culture; reasons for health choices; types of therapies used; barriers to use; and triggers or reasons for seeking this type of health care.^4^ However, without the proper research instrument specific to CAM, it is difficult to improve comparability between studies and continue to develop knowledge in this area, particularly if investigators want to move beyond the descriptive level.^4^,^9^

### Objective

The purposes of this study were 1) to develop and examine the psychometric properties of the CAM Questionnaire for Young Adults, which measures attitudes about CAM, and 2) to describe factors that are associated with young adults’ attitudes and possible intention to use CAM.

The questionnaire is not intended to measure outcomes of CAM use; rather it is designed to measure attitudes about CAM among adolescents and young adults. Accurately capturing attitudes about CAM will provide a better estimate of the likelihood to use CAM. This will, in turn, help to inform health care professionals about the level of demand for CAM in the young adult population; information that is currently not known. An appreciation of level of demand is particularly important when considering the need for integrative care models and studies aimed at utilization patterns.

## Method

### Design and sample

Participants for this cross-sectional survey were selected from enrolled undergraduate students within different age groups at an urban university. The sample size for preliminary psychometric testing of a newly developed instrument was based on the rule of thumb that the scale should be tested on 5–10 people per questionnaire item.^10^ The initial instrument contained 41 items; therefore a final sample of 205–410 was sought. Based on a conservative estimate of a 30% response rate, preparations were made to distribute the questionnaire to approximately 1500 adolescents and young adults, to yield a potential final sample of 450.

### Procedure

A student list was obtained from the University Registrar and categorized by year of birth and sex so that students from different age groups would be invited into the study. This was not possible for the youngest age group, birth year 1989, since there were too few candidates. Any records with incomplete data or addresses outside of Canada were excluded.

The survey was mailed to 1463 students. One hundred and seventy participants were systematically selected from each sex and age group by dividing the number of desired participants by the total number available, and using the result to invite every 10th person to complete the CAM Questionnaire (Table 1).

Each participant was assigned a unique ID that was used to track survey follow-up and for data entry. Surveys were mailed to the addresses on file and were returned directly to the investigators via a postage-paid self-addressed return envelope, which was included in the mailing package. Non-responders were followed up by e-mail a maximum of three times. The procedures followed were in accordance with the ethical standards of the Research Ethics Board (REB), McMaster University and ethics approval to conduct the study was received from the REB.

### Measures

The CAM Questionnaire, an initial 41 item scale, assessed respondents’ attitudes toward use of CAM. Respondents were asked to indicate their level of agreement with items on a 5 point Likert scale ranging from 1 = “strongly disagree” to 5 = “strongly agree.”

The content of the CAM questionnaire was generated using the results of a qualitative study of adolescent CAM users (n = 13) and a systematic review of the literature.^11^

During the development phase, the content of the scale was validated through expert review. The questionnaire was tested for clarity, comprehensibility and content with the input of the following individuals: a) a research director, b) a social worker involved with adolescents, c) naturopathic doctors and d) adolescents (n = 6). The penultimate scale was pilot tested on a group of adolescents (n = 32) to further ensure clarity and comprehensibility, specifically checking for any vague or value-laden terminology and length of items. Changes were made to any word or statement that was not clear. The total possible score for the initial CAM questionnaire ranged from 41 (negative attitude to use of CAM) to 205 (positive attitude toward use of CAM).

Attached to the CAM questionnaire was a background information sheet asking respondents for information about their age, sex, education, cultural and religious traditions, general health and their use of CAM.

### Statistical analysis

Responses to each survey item on the CAM questionnaire were graphed and the frequencies were examined. Following descriptive analysis, the data were examined for internal consistency by exploring item-total correlations. Those items that were found to have an item-total correlation less than 0.30 were excluded from further analysis. Remaining items were entered into a principal components factor analysis to identify subscales. On completion of the factor analysis, both the entire scale and the subscales were tested for internal consistency and Cronbach’s alpha was determined.

Frequencies were used to describe respondent background characteristics. Chi-square analysis was used to examine differences in likelihood to use CAM based on respondent characteristics; this was done using respondent total scale scores, divided at the median. An alpha level of 0.05 was considered significant for all tests.

## Results

### Respondent characteristics

As mentioned, the survey was mailed to 1463 students attending university in an urban centre. The response rate was 16% (n = 227).

Most survey respondents were female (68%) with a mean age of 19.5 (SD 1.29). The majority of responders were CAM users (72%, n = 164). Respondents used CAM for preventing and treating illness almost equally. A larger percentage of CAM users were female (73%, n = 120) than male (27%, n = 44) (Table 2). The most frequently used therapies included vitamins and minerals, herbal medicine, massage, yoga, and chiropractic services. (Table 3).

### Analysis of the CAM questionnaire

Histograms of responses revealed a normal distribution so that it was appropriate to use the item mean and standard deviations to describe the results. Examining the item-total correlation revealed that 28 of the 41 items displayed a correlation that was less than 0.30. These items were excluded from further analysis.

Following item reduction, there were 13 remaining statements in the CAM questionnaire. With 13 items, the sample size of 227 was sufficient to proceed to analysis of the psychometric properties of the instrument. The 13 items were entered into a factor analysis to identify possible subscales of the CAM questionnaire. The number of factors was decided upon by eigenvalues greater than one, and confirmed by the scree plot. To obtain a clear picture of the factor loadings in the final solution, a varimax rotation was used. Three subscales were identified explaining 50% of the variance in likelihood to use CAM. The subscales are:

1. Positive beliefs about CAM, which assessed young adults’ attitudes about the benefits of CAM. Items in this subscale referred to information, less side effects, natural products, access, empowerment and self-healing.
2. Environmental influence, which included the knowledge young adults have about CAM treatments, if their parents or family use CAM, if their friends used CAM and if their coaches or teachers discuss CAM with them.
3. Psychological comfort represents the young adults’ reasoning behind their attitudes toward CAM; such that it incorporates physical, mental and spiritual aspects of health. CAM may be used by those who fear the discomfort of medical treatments and believe that using CAM is not harmful.

Reliability analysis of the reduced CAM questionnaire showed that the overall Cronbach’s alpha was 0.79 with the following subscale alphas: 0.75 positive beliefs about CAM; 0.63 environmental influences; 0.51 psychological comforts (Table 4).

Subscales:

- Positive beliefs about CAM: Items 1–6
- Environmental influences: Items 7–10
- Psychological comfort: Items 11–13

The CAM Questionnaire was also tested for convergent validity by correlating the results with the Holistic Complementary and Alternative Medicine Questionnaire (HCAMQ), which was administered to a small sub-sample of respondents (r = −0.326, p = 0.056, N = 35). The HCAMQ is an 11 item scale, six of the items relate to beliefs about the scientific validity of complementary and alternative medicine and five to beliefs about holistic health. The scale was tested on rheumatology outpatients who mean age in years were 52.

The correlation between these two scales approached significance, supporting the notion that the two instruments are generally measuring the same underlying construct. Similarly the CAM Questionnaire was tested for discriminant validity with the Leeds Situational Anxiety Scale (r = 0.307, p = 0.093, N = 31). A non-significant result was found, which would be the expected outcome if, as anticipated, the scales are measuring different constructs.

### CAM questionnaire

Young adults’ attitudes toward the use of CAM were determined first by computing a total score on the 13-item CAM questionnaire. The mean total score was 45.8 (SD = 5.96) out of a possible 65.

To examine the association of the CAM Questionnaire score with respondents’ demographic characteristics, the total score was dichotomized at the median.^46^ Scores in the range of 0–46 were defined as negative attitude and low likelihood to use CAM, whereas scores between 47–65 were defined as positive attitude and higher likelihood to use CAM.

Young adults demonstrated a higher likelihood to use CAM if they were female, (Chi-square = 9.69; p = 0.003); used CAM for preventing illness in the past (Chi-square = 12.3, p = 0.001); or used CAM for treating illness in the past (Chi-square = 11.89, p = 0.001). They demonstrated a lower likelihood to use CAM if they had never used CAM, (Chi-square = 7.26, p = 0.007).

## Discussion

While other authors have reported that adolescents and young adults use CAM therapies in managing their health,^5^–^8^,^12^–^16^ none have used a validated scale that is specific to this population to achieve their findings. This study provides preliminary evidence in support of a measurement tool for use in studies of CAM in young adults. The CAM Questionnaire is short, easy to administer, has good internal consistency and, in this study, shows beginning demonstration of validity.

Aside from the psychometric properties of the scale, its use in this sample revealed that young adults who are female and have used CAM in the past for preventing or treating illness have the most positive attitude toward CAM and the greatest likelihood to use CAM again. Young adults who had never used CAM displayed the lowest attitude scores and therefore may be most unlikely to use it in the future. The findings related to females having a more positive attitude towards CAM differ from those reported in an earlier qualitative study, where the influence of sex on CAM use was inconclusive.^10^ In this study, some adolescents believed that females were more willing to try CAM because they are more conscious of their health, serious and open-minded. A few of the adolescents stated that males were more likely to want a ‘quick fix’ to a health care problem. Others disagreed; they believed that gender was not a factor in influencing CAM use at all. Social influencing factors, such as friends^7^,^12^ and parents or family members^5^,^7^ have been reported in other studies and are consistent with the results of the factor analysis on the CAM questionnaire.

The presence of illness has been identified as a contributor to CAM use in many studies.^6^,^5^,^13^,^16^ Participants in these studies were more likely to use CAM if they experienced frequent symptoms^5^ or had longer duration of illness.^6^ There is no reported evidence for the use of CAM therapies by adolescents and young adults for preventive health care. However, factor analysis of the CAM Questionnaire suggests that prevention may play an important role in young adults’ attitudes about CAM. This is a potential focus for future research.

The three subscales that emerged from the analysis can also be supported in the literature: 1) positive beliefs, 2) environmental influences, and 3) psychological comfort. Perceived benefits of using CAM is supported by 5 of the 9 studies found in the systematic review. Participants used CAM because of perceived efficacy,^7^,^12^,^16^ because it is natural,^7^,^12^ easy to get^7^,^12^,^16^ and because people around them advise them to do so.^12^,^16^ Adolescents also perceived that CAM is less harmful than modern Western medicine, and without side effects.^16^ Furthermore, from qualitative interviews^11^ it was found that family, teachers and friends had an influence on the adolescent’s use of CAM. The majority of adolescents agreed that their friends were influential in their decision to use CAM. Friends played a role in providing assistance with uncertainty around decision-making and telling about a success of treatment for a problem. CAM was also promoted when a CAM provider was a friend. Family was generally seen as an influencing factor for use by adolescents. Some parents influenced use by exposing adolescents through their personal use of CAM or supporting the decision to try it. Some adolescents believed that the support for CAM by parents is rooted in personal beliefs regarding effectiveness, traditions and natural versus chemical approaches to health. Finally, the psychological benefits involve the mind-body connection and its association with health. The 3-factor solution that emerged in the factor analysis of the CAM Questionnaire is reflective of a number of influences that have been previously reported in qualitative or descriptive studies. The emergence of these factors in a psychometric analysis where items are based on prior published work, strengthens the case for these being the important attitudinal contributors to CAM use by young adults.

Potential limitations to this study include the large number of non-responders and the fact that the majority of survey responders were female and were already CAM users. A possible reason for the response rate is the fact that the questionnaire was sent by mail, in paper-based format. A number of respondents suggested that an electronic format would have been preferable; this reflects the age and technology-based characteristics of a young adult sample. Future studies should focus on testing the instrument on a larger sample, with a specific effort to include younger adolescents, and should seek to sample equally from both males and females to avoid a gender bias. Since this questionnaire was specifically designed with a focus on adolescents and young adults, we would not recommend its use in other age groups without modification of the questionnaire items. Such modification would require further psychometric testing. In addition, there should be investigation of whether the questionnaire is applicable across ethnic and social status groups. However, the psychometric properties found in this study show promise for use of the CAM Questionnaire in practice and research. It also addresses the need for more CAM-specific instruments and may be the first of its kind for assessing adolescents’ and young adults’ attitudes toward use of CAM.

## Figures and Tables

**Table:** 

		**Strongly Disagree**	**Disagree**	**Haven’t decided**	**Agree**	**Strongly agree**
1	CAM providers give good information on maintaining a healthy lifestyle	1	2	3	4	5
2	There are less side effects when taking natural remedies	1	2	3	4	5
3	CAM involves natural plant formulas which are more healthy than taking drugs given by the medical doctor	1	2`	3	4	5
4	Young adults would be more likely to use CAM if there were more CAM clinics	1	2	3	4	5
5	Young adults are more empowered when using CAM because CAM providers involve them in decisions about their health care treatments	1	2	3	4	5
6	Young adults believe that CAM builds up the body’s own defenses and promotes self-healing	1	2	3	4	5
7	The more knowledge a young adult has about CAM, the more likely he/she is to use it	1	2	3	4	5
8	Parent(s) and family can influence a young adult’s CAM use by exposing them to it	1	2	3	4	5
9	Young adults are more likely to use CAM if their friends are using it	1	2	3	4	5
10	Young adults are more likely to use CAM if coaches and teachers discuss it with them	1	2	3	4	5
11	Young adults who believe in the physical, mental and spiritual aspects of health are more likely to use CAM	1	2	3	4	5
12	Young adults who fear the discomfort of treatments from medical doctors are more likely to use CAM	1	2	3	4	5
13	Young adults believe that taking CAM therapies is not harmful	1	2	3	4	5

**Table 1:** Tracking of participants.

**Number of participants**	**Year of birth**					**Total**
	**1989**	**1988**	**1987**	**1986**	**1985**	
Total	108	3484	3899	3273	2830	13594
Incomplete Data/outside Canada	5	50	53	60	61	229
Sent the CAM Questionnaire						
Males	49	170	170	170	170	729
Females	54	170	170	170	170	734
Responded						
Males	3	15	16	22	18	74
Females	11	33	27	38	44	153

**Table 2:** Respondent characteristics (n = 227).

**Characteristics**	**n (%)**
Gender	
Male	74 (33)
Female	153 (67)
Age	
17	12 (5)
18	51 (22)
19	39 (17)
20	59 (26)
21	63 (29)
22	3 (1)
Use CAM for*:	
Preventing illness	105 (46)
Treating illness	95 (42)
I have never used CAM	63 (28)

* Respondents may have chosen both preventing and treating illness.

**Table 3:** CAM product or therapy used in the past.

**Rank**	**Product or therapy**	**Total**
1	Vitamins and Minerals	122
2	Herbal Medicine	76
3	Massage	63
4	Yoga	46
5	Chiropractic	41

* Note that respondents were able to endorse more than one response option.

**Table 4:** Complementary and alternative medicine questionnaire.

**Number item**	**Item**	**Factor loading**	**Mean (SD)**
1	CAM providers give good information on maintaining a healthy lifestyle	0.59	3.47 (0.76)
2	There are less side effects when taking natural remedies	0.72	3.10 (1.09)
3	CAM involves natural plant formulas which are more healthy than taking drugs given by the medical doctor	0.74	2.91 (1.07)
4	Young adults would be more likely to use CAM if there were more CAM clinics	0.39	3.50 (0.88)
5	Young adults are more empowered when using CAM because CAM providers involve them in decisions about their health care treatments	0.61	3.30 (0.80)
6	Young adults believe that CAM builds up the body’s own defences and promotes self-healing	0.70	3.59 (0.80)
7	The more knowledge a young adult has about CAM, the more likely he/she is to use it	0.65	3.79 (0.91)
8	Q14 Parent(s) and family can influence a young adult’s CAM use by exposing them to it	0.73	4.18 (0.63)
9	Young adults are more likely to use CAM if their friends are using it	0.60	3.64 (0.92)
10	Young adults are more likely to use CAM if coaches and teachers discuss it with them	0.65	3.76 (0.71)
11	Young adults who believe in the physical, mental and spiritual aspects of health are more likely to use CAM	0.73	3.71 (0.88)
12	Young adults who fear the discomfort of treatments from medical doctors are more likely to use CAM	0.79	3.42 (0.91)
13	Young adults believe that taking CAM therapies is not harmful	0.39	3.39 (0.83)

## Complementary and Alternative Medicine Questionnaire

Please read this introduction carefully, as it will help you answer the questions.

Thank you for taking the time to complete this questionnaire. The questionnaire is designed to examine use of complementary/alternative health therapies.

Complementary/alternative medicine (or CAM) is defined as any natural health products (example, herbal products, vitamins, minerals, traditional Chinese medicine) or therapies (example, acupuncture, naturopathy, homeopathy, chiropractic manipulation, yoga, reflexology) you use or have used that were NOT suggested by a medical doctor. Generally, other health care practitioners such as Naturopaths, Traditional Chinese practitioners and Chiropractic practitioners provide CAM.

***Complementary*** means that you would use treatments suggested by a complementary/alternative provider **at the same time** as treatments suggested by a medical doctor.

***Alternative*** means that you would use complementary/alternative treatments **instead of** treatments that would be suggested by a medical doctor.

The questionnaire is divided into three parts.

**Part A: Asks information about you.**

**Part B: Asks information about therapies and providers that you have used.**

**Part C: Asks information about your beliefs towards complementary/alternative medicine (CAM) use.**

## Instructions

### Part A: Information about you

Please respond to the following questions. If you have additional comments about a specific question, print them in the space provided at the end of the question or questionnaire.

1) What is your age?_________________
2) Sex:     □ male       □ female
3) What grade have you completed?
  1 □ high school Grade 9
  2 □ high school Grade 10
  3 □ high school Grade 11
  4 □ high school Grade 12
  5 □ College or Institute Year 1
  6 □ College or Institute Year 2
  7 □ College or Institute Year 3
  8 □ College or Institute Year 4
  9 □ University Year 1
  10 □ University Year 2
  11 □ University Year 3
  12 □ University Year 4
  13 □ Other PLEASE SPECIFY: ______________________________________
4) What cultural traditions influence your decisions about health care? **(Check all that apply)**
  1 □ Chinese
  2 □ Canadian
  3 □ Italian
  4 □ Asian
  5 □ British
  6 □ Irish
  7 □ Greek
  8 □ African
  9 □ Aboriginal
  10 □ French
  11 □ Thai
  12 □ American
  13 □ Native American
  14 □ Norwegian
  15 □ Argentine
  16 □ Australian
  17 □ Lebanese
  18 □ Pacific Islander
  19 □ Other ___________
  20 □ No Influence
5) What religious traditions influence your decisions about health care? **(Check all that apply)**
  1 □ Muslim
  2 □ Judaism
  3 □ Catholic
  4 □ Buddhism
  5 □ Anglican
  6 □ Mormonism
  7 □ Islam
  8 □ Hinduism
  9 □ Christian Science
  10 □ Jehovah’s Witness
  11 □ Spiritualism
  12 □ United Church of Canada
  13 □ Other Christian denominations
  14 □ Other ___________
  15 □ No Influence
6) In general, would you say your health is:
  1 □ excellent
  2 □ very good
  3 □ good
  4 □ fair
  5 □ poor

### Part B: Therapies and providers that you have used

7) Have you used complementary/alternative medicine (CAM) for: **(Check all that apply)**
  1 □ preventing illness
  2 □ treating illness
  3 □ promoting health
  4 □ I have ***never used*** CAM
  5 □ Other________________________
  If you answered that you have **never used CAM**, skip to **Part C.**
8) Identify the statement that best describes your health care practices:
  1 □ I use CAM only
  2 □ I use CAM with treatments given to me by my medical doctor
9) Which natural health products and therapies do you use or have you used in the past? **(Check all that apply)**
  1 □ Acupuncture
  2 □ Acupressure
  3 □ Aromatherapy
  4 □ Art therapy
  5 □ Ayurveda
  6 □ Bach Flower remedies
  7 □ Chiropractic
  8 □ Colour therapy
  9 □ Dance Movement therapy
  10 □ Spiritual Healing
  11 □ Herbal medicine
  12 □ Homeopathy
  13 □ Hypnosis
  14 □ Magnetic therapy
  15 □ Massage
  16 □ Meditation
  17 □ Transcendental Meditation
  18 □ Music therapy
  19 □ Naturopathy
  20 □ Osteopathy
  21 □ Reiki
  22 □ Reflexology
  23 □ Relaxation/Breathing technique
  24 □ Shiatsu
  25 □ Traditional Chinese Medicine
  26 □ Therapeutic Touch
  27 □ Visualization
  28 □ Vitamins and minerals
  29 □ Yoga
  30 □ Other ____________________
10) Identify the statement that best describes your intake of natural health products. A natural health product includes ***vitamins and minerals*. (Check only one box)**
  1 □ I do not take natural health products
  2 □ I take natural health products on a daily basis
  3 □ I take natural health products on a weekly basis
  4 □ I take natural health products on a monthly basis
  5 □ I take natural health products once a year
  6 □ I take natural health products less often than once a year
  7 □ Other_________________________________
11) Identify the statement that best describes your level of involvement with a CAM provider. **(Check only one box)**
  1 □ I do not see CAM providers
  2 □ I see CAM providers on a daily basis
  3 □ I see CAM providers on a weekly basis
  4 □ I see CAM providers on a monthly basis
  5 □ I see CAM providers once a year
  6 □ I see CAM providers less than once a year
  7 □ Other_________________________________

### Part C: Your beliefs about CAM use

**In this section, young adult is an individual between the ages of 15 and 21 years of age.**

Listed below are a number of statements concerning your beliefs about CAM use? For each statement you should circle the number that corresponds most closely to your belief. **Circle only one number. Please do not miss any statements.**

Thank You

## References

[1] DiClemente RJ (1999). The psychological basis of health promotion for adolescents. Adolescent Medicine.

[2] Byrnes JP (2002). The development of decision-making. Journal of Adolescent Health.

[3] Noone J (2002). Concept analysis of decision making. Nursing Forum.

[4] Braun CA, Halcon LL, Bearinger LH (2000). Adolescent use of alternative and complementary therapies: A framework for research. Journal of Holistic Nursing.

[5] Reznik M, Ozuah PO, Franco K, Cohen R, Motlow F (2002). Use of complementary therapy by adolescents with asthma. Archives of Pediatrics and Adolescent Medicine.

[6] Hagen LE, Schneider R, Stephens D, Modrusan D, Feldman BM (2003). Use of complementary and alternative medicine by pediatric rheumatology patients. Arthritis and Rheumatis.

[7] Wilson KM, Klein JD (2002b). Adolescents’ use of complementary and alternative medicine. Ambulatory Pediatrics.

[8] Wilson KM, Dryer HI, Sesselberg TS, Klien JD (2003a). Herbal medicine use by adolescents in the U.S.: Preliminary data and feasibility of online survey methods. Journal of Adolescent Health.

[9] Hyland ME, Lewith GT, Westoby C (2003). Developing a measure of attitudes: The holistic complementary and alternative medicine questionnaire. Complementary Therapies in Medicine.

[10] Norman GR, Streiner DL (1994). Biostatistics: The bare essentials.

[11] Patterson C, Arthur H, Noesgaard C (2007). Exploring adolescent complementary/alternative medicine (CAM) use in Canada. Journal of Interprofessional Care.

[12] Breuner CC, Barry PJ, Kemper KJ (1998). Alternative medicine use by homeless youth. Achieves of Paediatric Adolescent Medicine.

[13] Grootenhuis MA, Last BF, Graaf-Nijkerk JH, Van der WM (1998). Use of alternative treatment in pediatric oncology. Cancer Nursing.

[14] Lau JT, Yu A (2000). The choice between Chinese medicine and Western medicine practitioners by Hong Kong adolescents. American Journal of Chinese Medicine.

[15] Masatu MC, Klepp KI, Kvale G (2001). Use of health services and reported satisfaction among primary school adolescents in Arusha, Tanzania. Journal of Adolescent Health.

[16] Pommier J, Billot L, Mouchtouris A, Deschamps JP, Romero MI, Zubarew T (2002). French adolescent attitudes towards informal care for physical and emotional or relational problems. Acta Paediatrica.

